# Improving satellite-based PM_2.5_ estimates in China using Gaussian processes modeling in a Bayesian hierarchical setting

**DOI:** 10.1038/s41598-017-07478-0

**Published:** 2017-08-01

**Authors:** Wenxi Yu, Yang Liu, Zongwei Ma, Jun Bi

**Affiliations:** 1 State Key Laboratory of Pollution Control and Resource Reuse, School of the Environment, Nanjing University, Nanjing, Jiangsu 210023 China; 2 Department of Environmental Health, Rollins School of Public Health, Emory University, Atlanta, GA 30322 USA; 3 School of Geographic and Oceanographic Sciences, Nanjing University, Nanjing, Jiangsu 210023 China; 4 Jiangsu Collaborative Innovation Center of Atmospheric Environment and Equipment Technology (CICAEET), Nanjing, Jiangsu 210023 China

## Abstract

Using satellite-based aerosol optical depth (AOD) measurements and statistical models to estimate ground-level PM_2.5_ is a promising way to fill the areas that are not covered by ground PM_2.5_ monitors. The statistical models used in previous studies are primarily Linear Mixed Effects (LME) and Geographically Weighted Regression (GWR) models. In this study, we developed a new regression model between PM_2.5_ and AOD using Gaussian processes in a Bayesian hierarchical setting. Gaussian processes model the stochastic nature of the spatial random effects, where the mean surface and the covariance function is specified. The spatial stochastic process is incorporated under the Bayesian hierarchical framework to explain the variation of PM_2.5_ concentrations together with other factors, such as AOD, spatial and non-spatial random effects. We evaluate the results of our model and compare them with those of other, conventional statistical models (GWR and LME) by within-sample model fitting and out-of-sample validation (cross validation, CV). The results show that our model possesses a CV result (R^2^ = 0.81) that reflects higher accuracy than that of GWR and LME (0.74 and 0.48, respectively). Our results indicate that Gaussian process models have the potential to improve the accuracy of satellite-based PM_2.5_ estimates.

## Introduction

Particulate matter with aerodynamic diameters less than 2.5 μm (PM_2.5_) can penetrate into human lungs and bronchi; thus, they may lead to many adverse health effects^1, 2^. In recent years, particulate matter has become one of the most important air pollutants in China and has received considerable public attention^3^. In 2012, the Chinese government issued national PM_2.5_ standards and began to establish a ground-level PM_2.5_ monitoring network. In contrast to the unsatisfactory temporal and spatial coverage of ground-based monitoring sites, PM_2.5_ estimates derived from satellite-retrieved aerosol optical depths (AODs) can provide more detailed and comprehensive data support for further health-related research in both the spatial and temporal dimensions^4^. Of the many satellite AOD products, the AOD data retrieved by the Moderate Resolution Imaging Spectroradiometer (MODIS, http://modis.gsfc.nasa.gov) instrument aboard the Terra and Aqua satellites launched by the National Aeronautics and Space Administration (NASA) have been the most widely used.

To date, numerous studies have focused on constructing statistical relationships between satellite AOD retrievals and ground-level PM_2.5_ measurements that can then be used to estimate PM_2.5_ concentrations in places where AOD data are available. The statistical methods used in previous studies mainly include generalized linear regression models (GLMs)^5^, linear mixed effects (LME) models^6, 7^, geographically weighted regression (GWR) models^8–10^, generalized additive models (GAMs)^11^ or two-stage hierarchical models that include combinations of different statistical models^12, 13^.

Previous studies have determined that the relationship between PM_2.5_ and AOD values varies in space^14, 15^. GWR models can address the spatial variability and non-stationarity of regression parameters; thus, many studies have employed this method to address the spatial heterogeneity of the PM_2.5_-AOD relationship^8, 9, 16, 17^. Unlike traditional geostatistical methods, which rely on particular functions (such as wavelets and splines) to represent spatial relationships, Gaussian processes are one of the most intuitive methods to model spatial surfaces as realization of stochastic processes^18, 19^. Specifically, Gaussian processes consider the spatial effects as random variables by specifying their means and covariance functions, which is the major feature that distinguishes them from other traditional methods.

The hierarchical nature can help explain various sources of variations in PM_2.5_. In particular, our model can be described in the following three stages: for the first stage, PM_2.5_ concentrations are conditional on the distribution of AOD values, spatial and non-spatial random effects, which is the basic foundation of our model; the second stage mainly focuses on the distribution of spatial random effects, which are modeled by Gaussian processes with specific mean surface and covariance functions; the last stage concentrates on the conditional distribution of the covariance functions of Gaussian processes given by the hyperparameters we chose. This hierarchical approach is helpful when dealing with ambiguous variations^20^.

Comparatively, for GWR models, the coefficients of each independent variable (in our case, there is a single explanatory variable, AOD) and intercept are different at different locations, and the coefficients are intrinsically modeled as fixed numbers. In Gaussian processes settings, the AOD coefficient and the intercept (which are defined later as *β*_1_ and *β*_0_) remain the same in each daily mode, and it is the spatial random effect (defined later as *ω*_*i*_) that captures the geographical variations. Thus, compared to GWR, Gaussian processes separate out different sources of variation (the independent variable AOD, spatial random effects and non-spatial random effects) in explaining PM_2.5_. This feature can be invaluable in uncertainty assessment, although this aspect is not covered in this article^21, 22^. Notably, uncertainty analyses of PM_2.5_ exposures can be useful for future environmental health research. However, to date, few studies have developed Gaussian process models for PM_2.5_-AOD modeling.

Along with the advancement of Geographical Information Systems (GISs), an increasing number of studies in areas such as environmental science, epidemiology and health policy management are using large spatiotemporal datasets. Of the existing spatial statistical methods, Bayesian methods have gained in popularity because of its sound reasoning of treating parameters as random quantities rather than fixed values. Parameters are updated by calculating the posterior distribution (*prob*(*parameters*|*data*)) by the incorporated external knowledge with respect to the distribution of parameters and the likelihood function (*prob*(*data*|*parameters*)). The Bayesian methodology is flexible because it allows non-informative priors, as well as informative priors acquired by relevant research or spatial variogram analysis^23^.

In recent years, several studies have employed Bayesian methods to improve satellite PM_2.5_ modeling. For example, Chang *et al*. applied a unified Bayesian hierarchical framework to improve PM_2.5_-AOD modeling that allows the model to calculate the prediction uncertainties, which are invaluable in further health impact analyses^24^. However, to our knowledge, no published studies have captured the spatial effects as stochastic processes in the Bayesian hierarchical setting to enhance the modeling performance of PM_2.5_ values estimated from satellite data. Overall, the uniqueness of our model comparing to previous research lies in: (1) treating the spatial relationships as random variables, (2) enabling the explanation of multiple sources of variations and (3) flexible in including prior knowledge.

Additionally, fitting hierarchical models can be time-consuming owing to the large sample size and high cost of matrix decomposition, which is known as a “large-N” problem. However, the recent development of the R package “spBayes**”** has enabled researchers to construct multivariate Gaussian processes for point-referenced spatial models with high computational efficiency. This outcome is achieved by projecting the spatial random terms into a lower-dimensional subspace (i.e., performing dimension reduction)^20, 25^. In our study, the number of daily PM_2.5_ observations was acceptable, so we did not use dimension reduction. We believe that the concept of employing Gaussian processes in a Bayesian hierarchical framework has the statistical and computational potential to improve the performance of spatial PM_2.5_-AOD modeling.

In this article, we applied Gaussian processes using Bayesian computation methods to construct daily PM_2.5_-AOD models for China in 2013. We anticipate that our research will increase the accuracy of model fitting and cross validation and thus lead to more reliable predictions of PM_2.5_ concentrations in China. The spatial distribution and seasonal variations of PM_2.5_ in China in 2013 are also examined in this paper.

## Data and Methods

### Ground-level PM_2.5_ measurements

**Figure 1:**
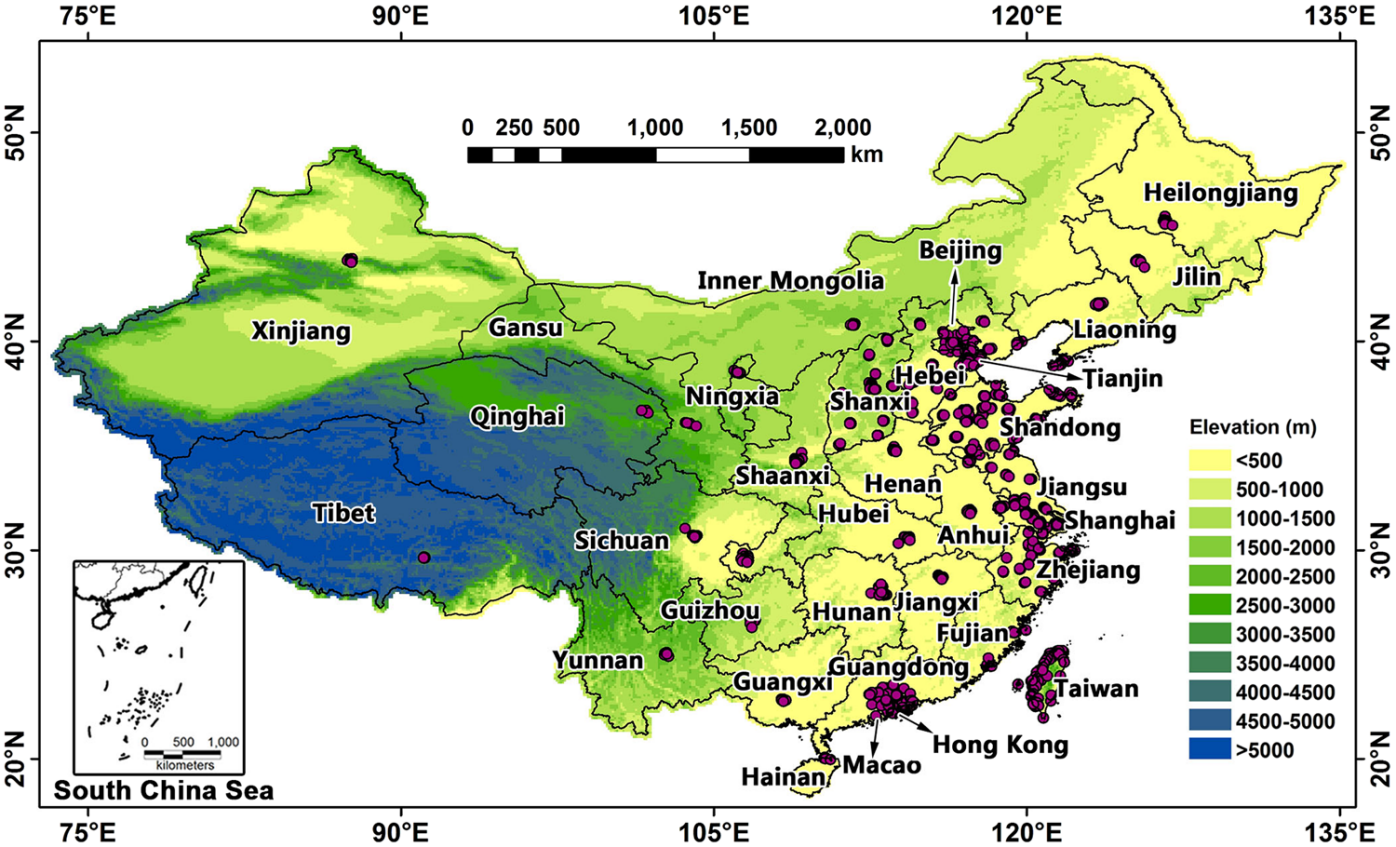
Spatial distribution of PM_2.5_ monitoring sites included this study. This map was generated using ArcGIS 9.3 (http://www.esri.com/software/arcgis)^26^ based on the geo-location information of the PM_2.5_ ground-based monitoring sites and elevation data. The elevation data were obtained from the Global Multi-resolution Terrain Elevation Data 2010 data product (https://lta.cr.usgs.gov/GMTED2010)^27^.

Daily average PM_2.5_ concentrations in China from Jan 1, 2013 to Dec 31, 2013 were primarily downloaded from the website of the China Environmental Monitoring Center (CEMC). We also collected PM_2.5_ data from additional ground-based monitoring sites that are not included in the CEMC database (including sites in the provinces of Shandong, Shanxi, Zhejiang and Guangdong and the cities of Beijing and Tianjin, as well as Macao, Hong Kong and Taiwan) from their official websites. We also obtained data from the U.S. consulate sites in Beijing, Shanghai, Guangzhou, Shenyang and Chengdu for use in this research. The ground-level PM_2.5_ concentrations were measured by Tapered Element Oscillating Microbalances (TEOM) or the beta-attenuation method. In summary, we obtained data from a total number of 840 sites located in 113 cities for this study. The spatial distribution of all of the ground-based monitoring sites is depicted in Fig. 1.

### Satellite AOD retrievals

In early 2014, NASA released the Aqua MODIS Collection 6 (C6) AOD products. These products include AOD data retrieved by the enhanced Dark Target (DT) and Deep Blue (DB) algorithms, which have been shown to be more accurate than previous versions of the DT and DB algorithms^28, 29^. The Aqua MODIS C6 data products also include an operational combined AOD product calculated from DB and DT AOD^28^. As the operational combined AOD data discards all DB AOD data with normalized difference vegetation index (NDVI) values > 0.3, which also has good performance, Ma *et al*.^13^ developed an inverse variance weighting (IVW) approach to combine the DT and DB AOD estimates. Their results show that the IVW-combined AOD has a performance that is comparable to that of the operational combined AOD values of MODIS, but it has a data coverage that is 90% greater. In this study, we used these IVW-combined AOD values as our AOD input dataset. A detailed description of the algorithm for generating IVW-combined AOD values can be found elsewhere^13^.

### Data processing

A 0.1° × 0.1° grid with 100,699 grid cells was created that covers all of China. The ground-level PM_2.5_ data were assigned to the corresponding grid cell by longitude and latitude. Following Ma *et al*.^13^, Thiessen polygons representing individual MODIS AOD pixels were created and overlapped with the grid to assign the IVW-combined AOD values to the grid cells. The ground-level PM_2.5_ data and the IVW AOD data were matched in those grid cells where both ground-level PM_2.5_ data and IVW AOD data were available to fit the model. Owing to the impact of clouds and coverage of the ground surface by snow and ice, the AOD coverage of the western parts of China (i.e., Tibet and Qinghai Province) is relatively low, causing a smaller sample size of PM_2.5_-AOD matchups in western China. For example, there are only 84 days with PM_2.5_-AOD matchups in Tibet. Our preliminary test shows that the small sample size in Western China could greatly affect the performance of our spatial model, which is similar to the conclusion reached in our previous study^17^. The main reason for this result is that we cannot determine the local relationship between the dependent and independent variables for Tibet for those days without PM_2.5_-AOD matchups. To address this problem, we followed Ma *et al*.^17^ in employing the Ordinary Kriging method to increase the sample size of PM_2.5_-AOD matchups. To ensure that we only interpolated AOD values that were spatially correlated with the IVW-combined AOD values, we conducted a variogram analysis to obtain the range values and then used the range values to create buffer zones for the grid cells where the IVW-combined AOD values were missing. If there were five or more grid cells with IVW-combined AOD values in the buffer zones, we then used interpolation to estimate AOD values for those grid cells that were lacking AOD values. The gridded PM_2.5_ data and interpolated AOD data were then matched by grid cell ID and Day of Year (DOY) for model development. This interpolation process increased the number of PM_2.5_-AOD matchups from 63,189 to 162,089. The interpolated AOD data are not used in the prediction process.

### Gaussian processes model development and validation

A separate PM_2.5_-AOD Gaussian process model was fitted for each day in this study. The basic daily spatial model can be described as

1 $$PM{2.5}_{i}={\beta }_{0}+{\beta }_{1}AO{D}_{i}+{\omega }_{i}+\varepsilon $$

where *PM*2.5_*i*_ (μg/m^3^) and *AOD*_*i*_ are the daily ground-level PM_2.5_ concentration and AOD value at location i, respectively; *β*_1_ and *β*_0_ are coefficients for *AOD*_*i*_ and the intercept, respectively, which are selected so that, for each daily model, *β*_1_ and *β*_0_ are consistent for all locations i; and *ω*_*i*_ and *ε* are terms that capture the spatial random effect and the random error, respectively. $\varepsilon  \sim N(0,{\tau }^{2})$, where *τ* ^2^ is called the nugget. *ω*_*i*_ is a multivariate Gaussian process (MVGP) with a distribution having a mean of 0 and covariance function of $K(h;\theta )$. That is, ${\omega }_{i} \sim MVGP(0,K(h;\theta ))$. We can further specify the covariance function by $K(h;\theta )={\sigma }^{2}\rho (h;\varphi ,\nu )$. *K* is the covariance function; *h* is the Euclidean distance between any two spatial locations; $\theta =({\sigma }^{2},\varphi ,\nu )$ denotes all of the parameters used in the covariance function; *σ* ^2^ is a variance parameter; $\rho (h;\varphi ,\nu )$ is the correlation function; *ϕ* is the spatial decay parameter ($\frac{1}{\varphi }$ stands for the effective range parameter, that is, the distance at which spatial correlations become negligible) and $\nu$ is the smoothness parameter that is often used in correlation functions, such as the Matérn correlation function. The spherical model^30^ was chosen as the spatial correlation model (see the Supplementary Information, Text S1) for use in this paper by conducting a daily variogram analysis of the PM_2.5_ concentrations and comparing the equally weighted ordinary least squares among four traditional methods (spherical, Matérn, linear and Gaussian). We note that the smoothness parameter *v* does not exist in the spherical model by definition. Therefore, we use $\rho (h;\varphi )$ and $\theta =({\sigma }^{2},\varphi )$ instead of $\rho (h;\varphi ,\nu )$ and $\theta =({\sigma }^{2},\varphi ,\nu )$ in the following discussion.

The spherical model can be summarized as follows.

2 $$\rho (h;\varphi )=\{\begin{array}{c}1-1.5h\varphi +0.5{(h\varphi )}^{3},0 < h < \frac{1}{\varphi }\\ 0\,otherwise\end{array}$$

We also defined the prior distributions for each parameter. Specifically, the mean parameters $\beta =({\beta }_{0},{\beta }_{1})$ follow normal distributions with assigned means and covariances. The variance parameters *τ* ^2^ and *σ* ^2^ both obey inverse gamma distributions with shape hyperparameters equal to 2 (thus, the variance is infinite, by definition), whereas the spatial decay parameter *ϕ* follows a uniform distribution. The reasons for selecting the corresponding prior distribution for each parameter are twofold. On the one hand, the type of distribution of each parameter was chosen by referencing previous studies^20^. On the other hand, some of the values of hyperparameters were selected so that each parameter has a broad range of potential values (greater variance), which allows for daily variations. The selection of prior distributions is mainly heuristic and subject to change. The parameters were updated using the Metropolis-Hastings algorithm. A summary of the prior distributions of all the parameters is presented in the Supplementary Information (Table S2).

We set the number of iterations for each parameter to 5,000. By monitoring the changes in these parameters, we found that they changed dramatically from the beginning (within 3,000 iterations) and gradually stabilized over time (See Supplementary Information, Text S2). Then, we recovered the regression coefficients *β* and spatial random effects *ω*_*I*_ from the parameters after a burn-in period of 3,000 iterations. With regard to the model fitting and cross-validation processes, we obtained the mean value of daily predictive PM_2.5_ according to the parameters of each iteration.

**Figure 2:**
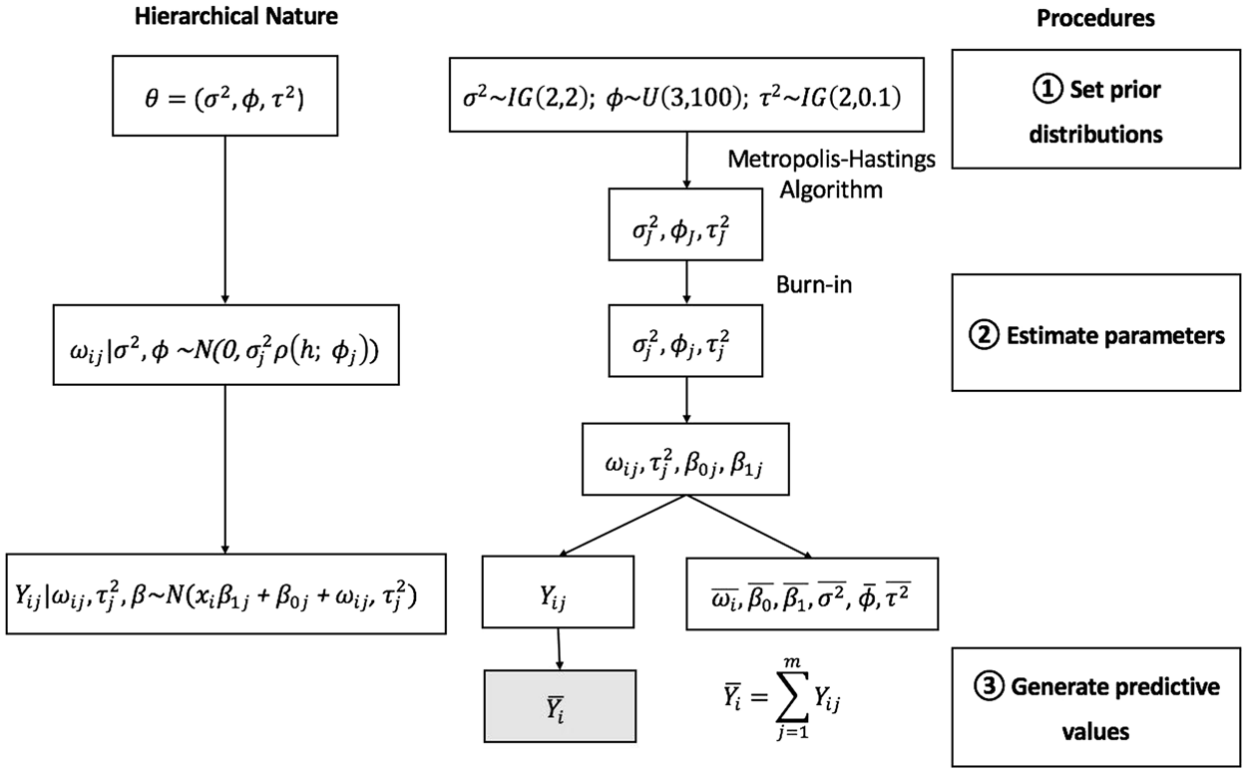
Hierarchical nature and procedures involved in constructing the daily Gaussian processes models. $i=\mathrm{1..}.,n$ is the location index ($n$ differs in each daily model); the iterations have indices $J=1,\mathrm{..}.,5,000$; after the burn-in period, only the iterations after 3,000 (including 3,000) are retained, so $j=\mathrm{1..}.,2001$. $\overline{{\omega }_{i}},\overline{{\beta }_{0}},\overline{{\beta }_{1}},\overline{{\sigma }^{2}}$, and $\bar{\varphi },\overline{{\tau }^{2}}$ are the daily mean values of each parameter. Other symbols have the same definitions as given previously.

The flowchart shown in Fig. 2 summarizes both the hierarchical nature and the overall procedure of selecting prior distributions, estimating parameters and generating predictive values. gray $\,\overline{{Y}_{i}}$ is the daily average of PM_2.5_ concentrations at location i, which is what we use in the further discussion.

We also provide a summary of the posterior distributions of the parameters in the Supplementary Information (Table S3). We first calculated the daily mean for each parameter by averaging the values after the burn-in period and then calculated the corresponding annual and seasonal averages.

In model fitting, the modeling dataset was used in both the model fitting and the model validation. This procedure does not account for the over-fitting problem; that is, the model might display better predictive performance for the dataset that was used to fit the model than in the rest of the data from the study area. In this study, 10-fold cross validation (CV)^31^ was used in the model validation process to avoid the obvious problem of over-fitting. In 10-fold CV, the modeling dataset is randomly split into 10 subsets of equal size. Of the 10 subsets, nine subsets are used for the model fit and the remaining subset is used to validate the model. The CV process is then repeated 10 times, and each of the 10 subsets is used exactly once for model validation. We compared the coefficient of determination (R^2^) and the root mean squared error (RMSE) from the model fitting and CV results. Finally, we applied the Gaussian processes model to estimate the PM_2.5_ concentrations in those grid cells where IVW AOD values were available.

### Comparisons with other statistical models

GWR and LME models are two statistical methods that are widely used in constructing relationships between PM_2.5_ and satellite AOD values. In this article, we compared the performance of our model with that of these two common methods by comparing the results of model fitting and 10-fold cross validation. Regarding the GWR model, the bandwidth of each daily model was selected by minimizing the result of leave-one-out cross validation and the geographical weighting function was constructed under Gaussian scheme^32, 33^. The daily GWR model for each day in 2013 can be described as follows:

3 $$PM{2.5}_{i}={\beta }_{0i}+{\beta }_{1i}AO{D}_{i}+{\varepsilon }_{i}$$

where $PM{2.5}_{i}$ and $AO{D}_{i}$ are the daily average values, respectively, at location i; *β*_o*i*_ and *β*_1*i*_ denote the intercept and slope, respectively, at location i; and *ε*_*i*_ is the error term for grid cell i. Thus at places where ground-level PM_2.5_ concentrations are not available, coefficients *β*_0*i*_ and *β*_1*i*_ are estimated by weighted least squares by taking distance and bandwidth into consideration.

Regarding the LME model,

4 $$PM{2.5}_{i,t}=(\mu +{\mu }_{t}^{^{\prime} })+(\beta +{\beta }_{t}^{^{\prime} })AO{D}_{i,t}+{\varepsilon }_{i,t} \sim N[(0,0),{\psi }_{1}]$$

where $PM{2.5}_{i,t}$ and $AO{D}_{i,t}$ are the PM_2.5_ concentration and the AOD value, respectively, at location *i* on day *t*; $\mu \,and\,{\mu }_{t}^{^{\prime} }$ are the fixed intercept and the random daily intercept, respectively; *β and* ${\beta }_{t}^{^{\prime} }$ are the fixed slope and daily random slope, respectively; and *ε*_s,t_ is the error term. In this LME model, the fixed effect represents the average intercept and linear PM2.5-AOD relationship for all study days. The random effects explain the daily variability of the intercept and slope of the linear PM2.5-AOD relationship^7^.

## Results and Discussion

### Descriptive statistics

Table 1 provides a summary of the descriptive statistics of the PM_2.5_ model dataset from annual and seasonal perspectives. The annual mean PM_2.5_ and AOD values are 67.9 µg/m^3^ and 0.77, respectively. Notably, winter is associated with the highest mean value of the daily PM_2.5_. Spring and fall share similar values, whereas summer is associated with the lowest daily average value. For AOD, the highest AOD values occur in winter, whereas spring, summer, and autumn share similar AOD levels.

**Table 1:** Summary statistics of the model dataset.

	Mean		Standard Deviation		Min		Max	
	PM_2.5_ (µg/m^3^)	AOD	PM_2.5_ (µg/m^3^)	AOD	PM_2.5_ (µg/m^3^)	AOD	PM_2.5_ (µg/m^3^)	AOD
Annual	67.9	0.77	60.6	0.63	0.63	−0.03*	902	4.38
Spring	58.2	0.76	40.1	0.50	3.00	0.01	407	3.75
Summer	42.8	0.77	33.5	0.67	1.62	−0.01	417	4.28
Autumn	65.5	0.76	50.4	0.66	2.43	−0.03	902	4.37
Winter	106	0.81	85.7	0.67	0.63	0.01	852	3.61

*Note that the MODIS Dark Target (DT) algorithm allows retrievals of small negative AOD values (down to −0.05)^28^.

### Gaussian processes model fitting and validation

**Figure 3:**
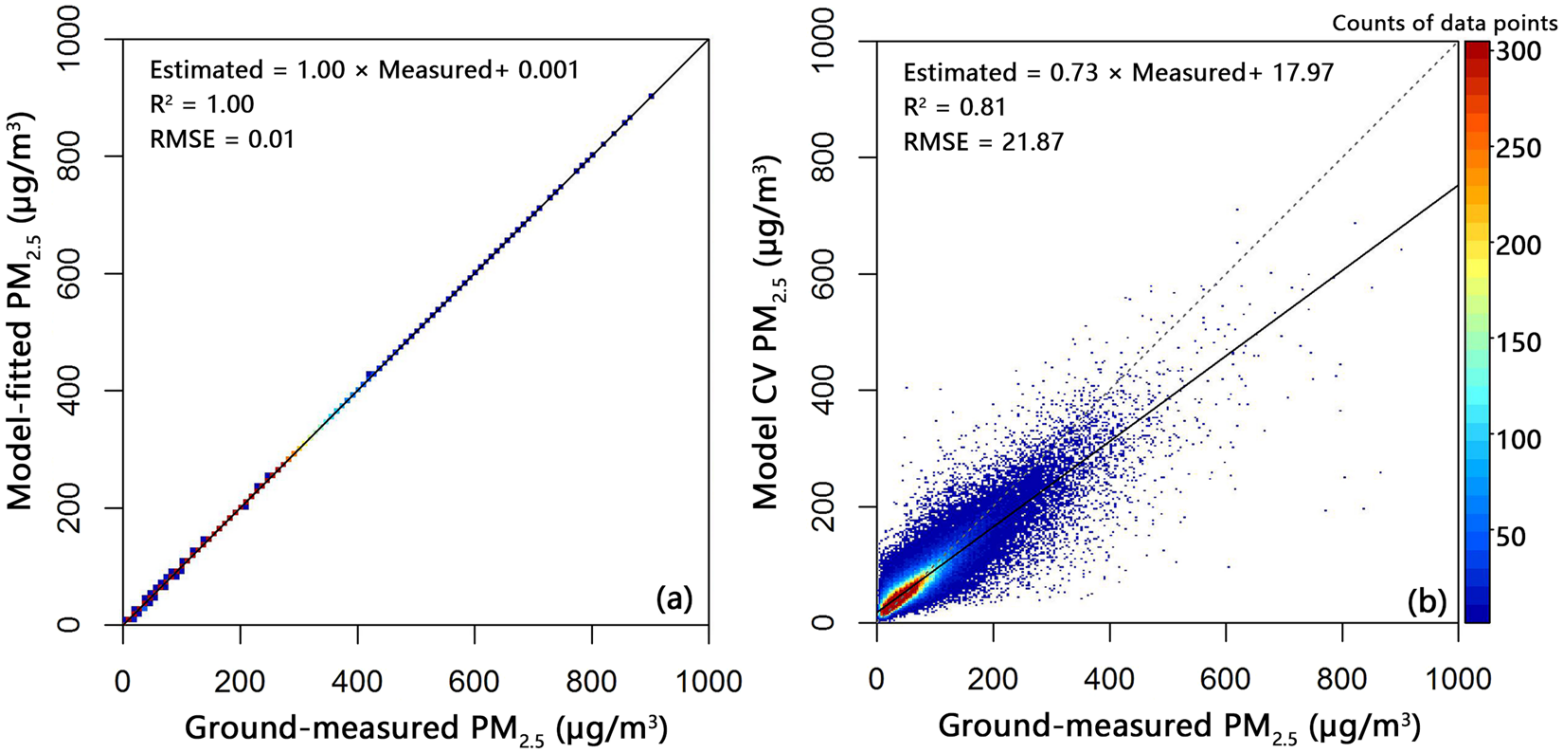
Model fitting (**a**) and cross validation (**b**) of the Bayesian Gaussian processes model (N = 162,089). RMSE: root mean squared prediction error (µg/m^3^). For reference, the 1:1 line is shown as a dashed line.

Figure 3 shows a scatterplot displaying the model fitting and 10-fold cross validation results of our Gaussian processes models. The R^2^ value for the model fit is very close to 1 because a large amount of the variation in PM_2.5_ is captured by our spatial random effects term *ω*_*i*_, which is generated from the posterior samples of *σ* ^2^ and *ϕ*. The RMSE value for the model fit is 0.01 µg/m^3^. For the model cross validation, the R^2^ and RMSE values are 0.81 and 21.87 µg/m^3^ respectively. This result shows that Gaussian processes in the Bayesian hierarchical setting may provide an improved description of daily spatial variations and generate more precise model results, given appropriate data. Regarding the R^2^ value, which is close to 1, similar results were found in a recent study^34^, which successfully developed a machine learning algorithm to estimate global PM_2.5_ values using satellite-based remote sensing data. They also obtained high model fitting correlation coefficients when training their models using the Terra and Aqua DB AOD datasets (both of which were associated with R^2^ = 1)^34^. However, the correlation coefficients of their model validation (R^2^ = 0.56 and 0.40 for Terra and Aqua DB AOD datasets, respectively) were lower than those of our Gaussian processes model (R^2^ = 0.81). Unlike model fitting procedures in which the entire dataset is used to both fit and validate the model, the cross-validation process does not use the PM_2.5_ data that have been used in the model fitting process to validate the model. Therefore, the cross-validation result will decrease compared to the model fitting result. In addition, in the application of this Gaussian processes model, the accuracy with which the PM_2.5_ values can be predicted in the grid cells where AOD values are available but ground-level PM_2.5_ values are unavailable will be close to our result from the cross validation (R^2^ = 0.81).

### Comparisons with other statistical models

**Figure 4:**
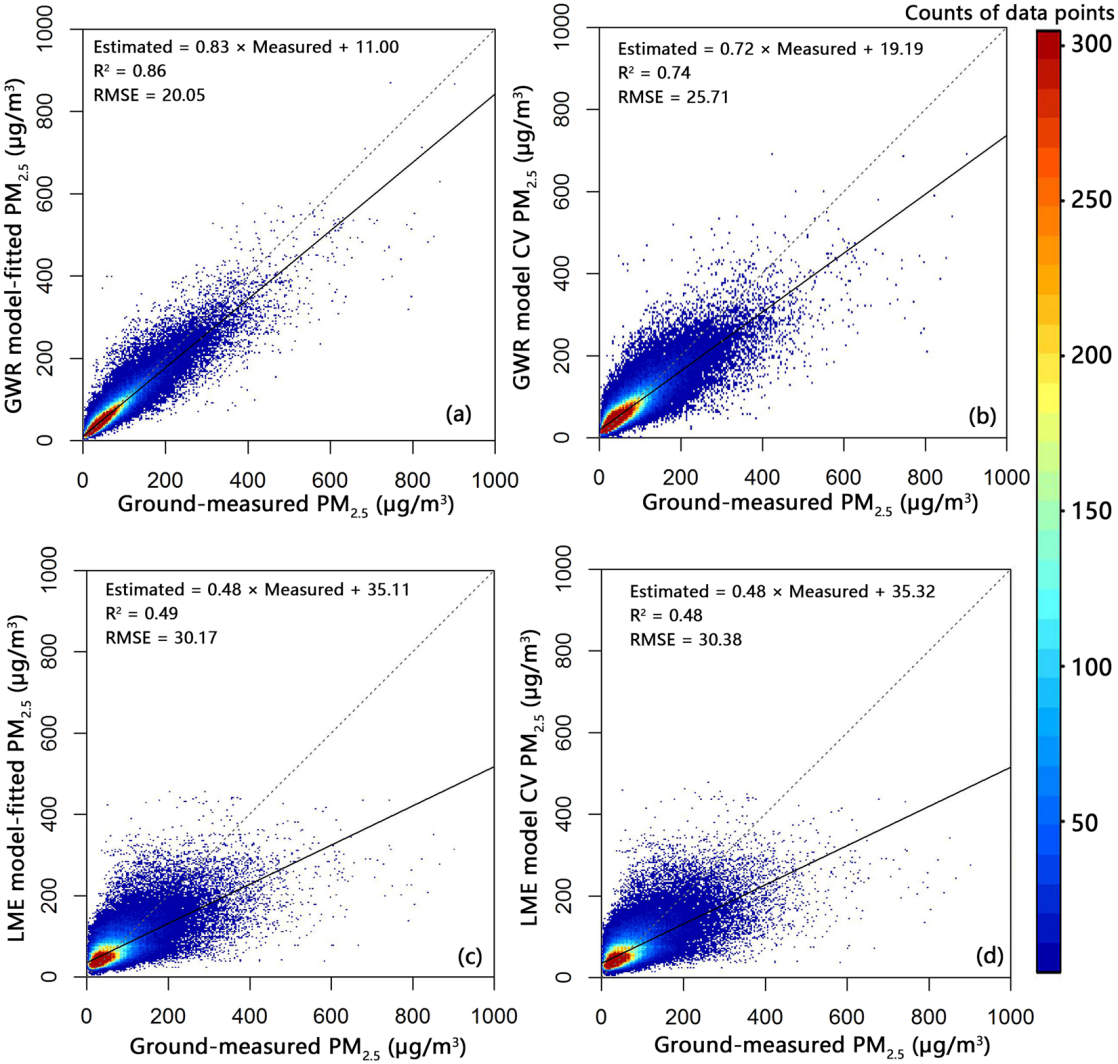
Model fitting and cross validation using GWR and the LME model (N = 162,089). RMSE: root mean squared error of the predictions (µg/m^3^). For reference, the 1:1 line is shown as a dashed line. Panels (a) and (b) show the model fitting and cross-validation results of the GWR model, whereas panels (c) and (d) show the model fitting and cross-validation results of the LME model, respectively.

In this study, we compared our results with those from other commonly adopted methods, specifically a Linear Mixed Effects model (LME) and Geographically Weighted Regression (GWR). Figure 4 presents the comprehensive model fitting and cross-validation results of these two models. Values of R^2^ that are close to 1 and small values of the RMSE suggest a more precise predictive ability of the model. Thus, GWR displays better performance in terms of its model fitting and cross-validation results than the LME model, as summarized in Table S5 (Supplementary Information).

Compared to GWR and LME, our Gaussian processes model is more strongly over-fitted; the value of R^2^ drops to a greater degree in the cross validation compared to the model fitting results (1–0.81 = 0.19), whereas the corresponding numbers for GWR and LME are 0.12 and 0.01, respectively. However, the Gaussian processes model obtains the highest cross-validation R^2^ of 0.81, which is much higher than those of the GWR model (cross-validation R^2^ = 0.74) and the LME model (cross-validation R^2^ = 0.48).

You *et al*.^16^ developed a national GWR model to estimate PM_2.5_ concentrations in China using MODIS and Multiangle Imaging Spectroradiometer (MISR) AOD products. They obtained cross-validation R^2^ values for MODIS and MISR AOD-based GWR models of 0.76 and 0.81, respectively, and these values are higher than those that we obtained with our GWR model and are comparable to those of our Gaussian processes model. However, this earlier study also included wind speed, air temperature, visibility, and relative humidity as covariates in their models. Previous studies have revealed that meteorological and land use variables can greatly improve PM_2.5_-AOD model performance^11, 35^. In our study, AOD is used as the sole explanatory variable. We expect that the performance of our Gaussian processes and GWR models could be improved if we incorporated meteorological and land use variables.

One previous study developed a two-stage spatial statistical model using MODIS AOD data, assimilated meteorological data, and land use data^13^. The first-stage of that model was an LME model, and the first-stage cross-validation R^2^ value was 0.79. This value is much higher than that of our LME model and approaches that of our Gaussian processes model. However, that study fitted the first-stage LME model for each province separately. They also emphasized that the cross-validation R^2^ of the first-stage LME model would drop to 0.63 if a single LME model was fitted for the whole of China^13^. In addition, their model performance could decrease further if the meteorology and land use variables were excluded. Comparing the GWR and LME models in our study and previous studies, our Gaussian processes model displays better model performance, and this method has the potential to improve the accuracy of satellite-based PM_2.5_ estimates.

### Spatial and seasonal variations in model-estimated PM_2.5_ concentrations

**Figure 5:**
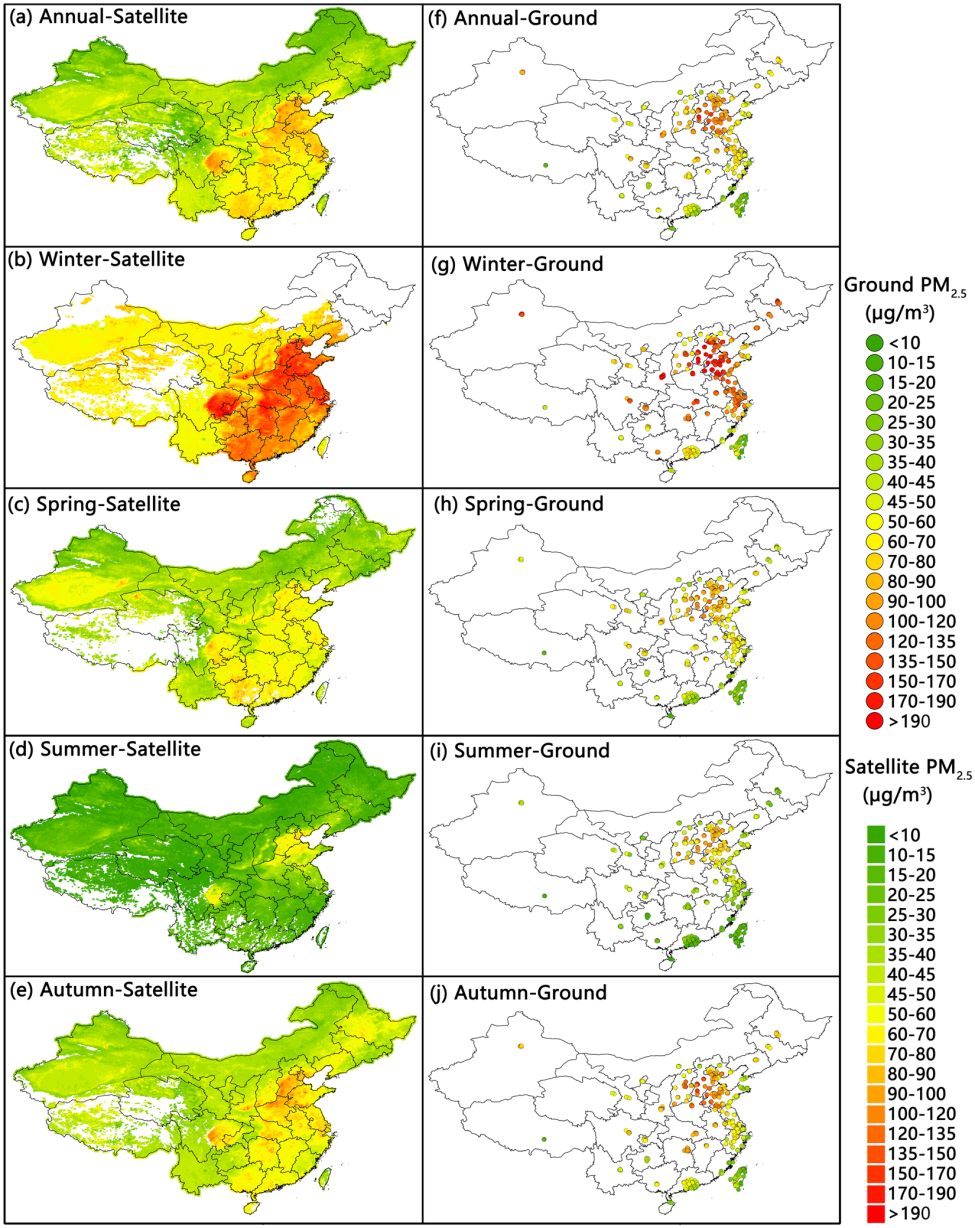
Seasonal and annual distributions of PM_2.5_ concentrations estimated using the Bayesian Gaussian process model. This map was generated using ArcGIS 9.3^26^ based on our Gaussian processes estimated PM_2.5_ data (left column) and the ground-level PM_2.5_ measurements (right column).

We applied the Gaussian processes model to the original IVW AOD data without first interpolating the data to generate daily PM_2.5_ concentrations for the year 2013. To compare the annual and seasonal variations in the PM_2.5_ concentrations, we averaged the result from each daily model on annual and seasonal bases. Figure 5 shows the annual and seasonal distributions of PM_2.5_ concentrations estimated by the Gaussian processes model (left column) and determined by ground-based measurements (right column). The left column reflects better spatial coverage because satellite-retrieved AOD values possess better spatial coverage than ground-based PM_2.5_ monitoring sites. Overall, the spatial patterns of our model-estimated PM_2.5_ concentrations are consistent with the data from the ground-based monitoring sites on both annual and seasonal scales. Specifically, the model predicted values substantially expand the area covered by valid PM_2.5_ data on the national grid compared to the ground-based air quality monitoring sites. The latter are mainly concentrated within the eastern coastal areas, whereas the former possess a more comprehensive coverage of the whole area of China. This result is consistent with previous PM_2.5_-AOD studies that indicated that AOD has a better spatial and temporal coverage than the ground-based monitoring network.

Regarding the variations in the seasonal averages, winter has the highest seasonal average PM_2.5_ value of all the seasons, with a value of 82.69 µg/m^3^. Spring and autumn have similar seasonal average values of 41.10 µg/m^3^ and 44.06 µg/m^3^, respectively, whereas summer has the lowest value of 16.52 µg/m^3^. There is also an apparent geographic variation among the different parts of China. The North China Plain and the Sichuan Basin, as well as central and eastern China, possess higher annual average PM_2.5_ values compared to other areas with better air quality, such as the northeastern and western parts of China. This spatial difference is consistent with the uneven distribution of socio-economic development; more developed areas may have higher annual average PM_2.5_ concentrations.

In summary, our Gaussian process model exhibits superior performance compared to two commonly used types of models (LME and GWR); it has more accurate predictive performance in model fitting and cross validation. Compared to the GWR model, the Gaussian process model increased the model cross-validation R^2^ value dramatically, from 0.74 to 0.81. Our model represents the first example of employing Gaussian processes in a Bayesian hierarchical setting to construct a spatial regression between PM_2.5_ and AOD. We hope that our preliminary study will play a part in stimulating further research and improving the prediction accuracy of future PM_2.5_-AOD modeling studies.

We did not include meteorological parameters and land use information in our Gaussian process model, which is a limitation that needs to be further examined in our future research work. However, the aim of this study is to examine the feasibility of producing improved PM_2.5_-AOD models using Gaussian processes. In addition, the results indicate that our model can potentially improve the accuracy of satellite-based PM_2.5_ modeling. It is expected that the Gaussian process model can be further improved if meteorological and land use variables are included.

## Electronic supplementary material

Supplementary Information
