# Supplementary material for: Improving satellite-based PM2.5 estimates in China using Gaussian processes modeling in a Bayesian hierarchical setting
Source: Sci Rep. 2017 Aug 1;7:7048. doi: 10.1038/s41598-017-07478-0 (PMC5539114; doi:10.1038/s41598-017-07478-0)
Supplement: Supplementary file 1 — Supplementary Information [file 41598_2017_7478_MOESM1_ESM.pdf]

Supplementary Information

Improving satellite-based $PM_{2.5}$ estimates in China using Gaussian processes modeling in a Bayesian hierarchical setting

Wenxi Yu [1], Yang Liu [2], Zongwei Ma [1,3,*], Jun Bi [1,4,*]

[1] State Key Laboratory of Pollution Control and Resource Reuse, School of the Environment, Nanjing University, Nanjing, Jiangsu, 210023, CHINA

[2] Department of Environmental Health, Rollins School of Public Health, Emory University, Atlanta, GA, 30322, USA

[3] School of Geographic and Oceanographic Sciences, Nanjing University, Nanjing, Jiangsu, 210023, CHINA

[4] Jiangsu Collaborative Innovation Center of Atmospheric Environment and Equipment Technology (CICAEET), Nanjing, Jiangsu, 210023, China

*Correspondence to:

Dr. Zongwei Ma, School of the Environment, Nanjing University, 163 Xianlin Avenue, Nanjing 210023, P. R. China. Tel.: +86 25 89681526. E–mail address: njumazw@163.com;

Dr. Jun Bi, School of the Environment, Nanjing University, 163 Xianlin Avenue, Nanjing 210023, P. R. China. Tel.: +86 25 89681605. E–mail address: jbi@nju.edu.cn.

Text S1: Selection of correlation functions

The choice of spherical model plays an important role in our daily spatial modeling. We selected the spherical model because it provides the best fit to most of the daily variogram plots when compared to the three other correlation models that we examined (Matérn, linear and Gaussian). Specifically, we calculated the equally weighted loss function for each model and found that for 58% of the days in a year (212 days) the spherical correlation function provides the best fit, whereas the Gaussian function provided the best fit on 34% of the days in the same year (124 days).

We subsequently found that even on days (take the day of the year=18 as an example; Table S1) when a variogram analysis suggested the use of the Gaussian function over the spherical function the model performance did not vary strongly, regardless of which correlation functions was chosen (for both, R^2=1). For consistency, we used the spherical correlation function for all of the daily models.

Table S1. Comparison of Gaussian and spherical correlation functions (day of year: 18)

		Gaussian	Spherical
Parameter estimates: 50% (2.5%, 9.5%)	β_0	62.70<br>(62.35, 63.06)	59.21<br>(58.89, 59.52)
	β_1	84.28<br>(83.87,84.69)	87.69<br>(87.32, 88.06)
	σ^2	4111.98<br>(4099.24, 4124.72)	3256.48<br>(3247.58, 3265.37)
	τ^2	0.05<br>(0.05, 0.05)	0.06<br>(0.06, 0.06)
	ϕ	6.02<br>(6.01, 6.02)	3.01<br>(3.01, 3.02)
Model performance (Model fitting)	R^2	1	1

Table S2. Prior distributions of all the parameters used in the Bayesian Gaussian processes model

Notation	Meaning	Type of distribution	Hyperparameters
$\boldsymbol{\beta}$	Mean parameter	Normal distribution	mean=0, covariance=$\begin{pmatrix} 1000 & 0 \\ 0 & 1000 \end{pmatrix}$
$\boldsymbol{\tau^2}$	Variance parameter (non-spatial nugget)	Inverse gamma distribution	shape=2, scale=0.1
$\boldsymbol{\sigma^2}$	Variance parameter (spatial)	Inverse gamma distribution	shape=2, scale=2
$\boldsymbol{\phi}$	Decay parameter	Uniform distribution	a=3, b=100

Table S3. Annual and seasonal averages of posterior samples (mean and 95% confidence intervals)

	β_0	β_1	σ^2	τ^2	ϕ
Annual	37.76 (35.75, 39.76)	37.60 (34.84, 40.37)	1226.73 (1080.06, 1373.4)	0.09 (0.09,0.10)	6.66 (5.41,7.91)
Spring	34.8 (31.67, 37.93)	29.65 (24.38, 34.92)	655.53 (548.13, 762.92)	0.10 (0.09,0.11)	7.06 (4.63, 9.49)
Summer	20.67 (19.19, 22.14)	29.33 (26.68, 31.98)	468.04 (423.2, 512.88)	0.09 (0.08, 0.09)	7.00 (4.15, 9.86)
Autumn	40.67 (37.78, 43.57)	32.99 (28.41, 37.58)	1043.17 (873.67, 1212.66)	0.09 (0.09, 0.10)	7.34 (4.62, 10.05)
Winter	55.3 (50.91, 59.68)	58.84 (52.16, 65.52)	2771.79 (2356.54, 3187.03)	0.10 (0.09, 0.011)	5.23 (3.21, 7.24)

Text S2: Selection of the number of iterations and burn-in times

We plotted the changes in all of the parameters over time to determine the appropriate number of iterations and burn-in times. Taking one daily model as an example (day of the year=18), we set the number of iteration (n.samples) equal to 500, 5,000 and 50,000 to see how each parameter would react using the Metropolis-Hastings algorithm (Figure S1-S3). Notably, even though σ^2 (sigma.sq) seemed to remain stable after 500 iterations, it still dropped drastically after 1,000 iterations, which clearly meant that 500 iterations were not sufficient for the parameters to converge. When we compared the results for 5,000 iterations with those obtained for 50,000 iterations, we found that all the parameters remained steady after 5,000 iterations. Thus, the use of 50,000 iterations will not lead to a substantial increase in stability; moreover, it will require much larger amounts of computational time and hinder the further application of this method for large datasets. We also note that setting the burn-in time equal to 3,000 should be sufficient to discard the unstable changes in the parameters and preserve the corresponding values after convergence.

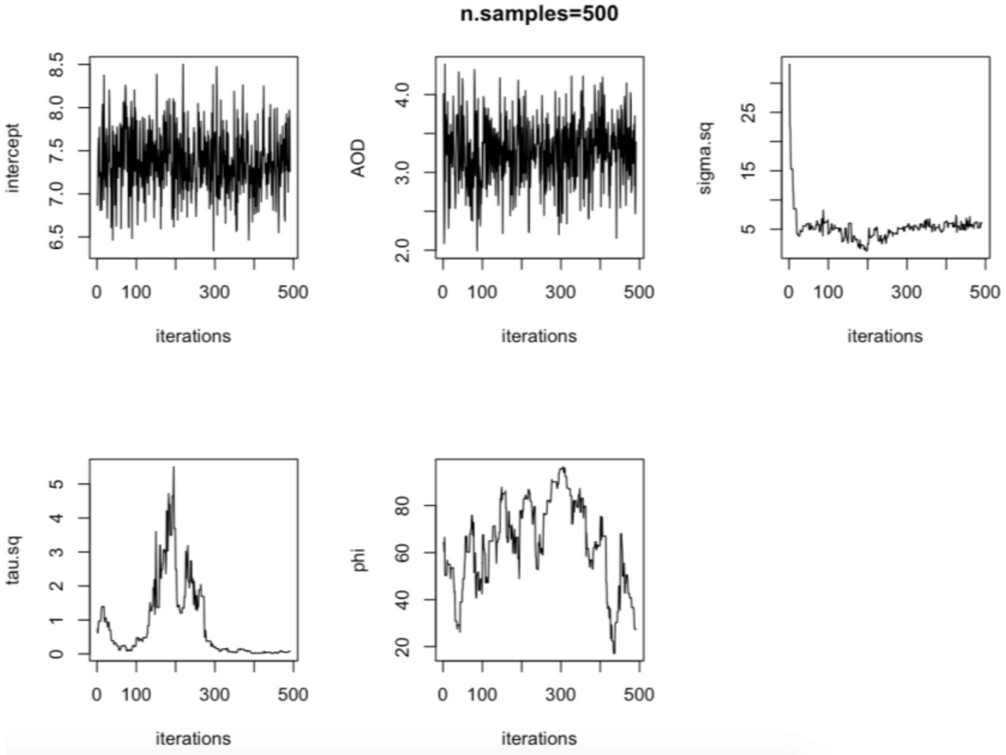

Figure S1. Trace plots of all the parameters after 500 iterations.

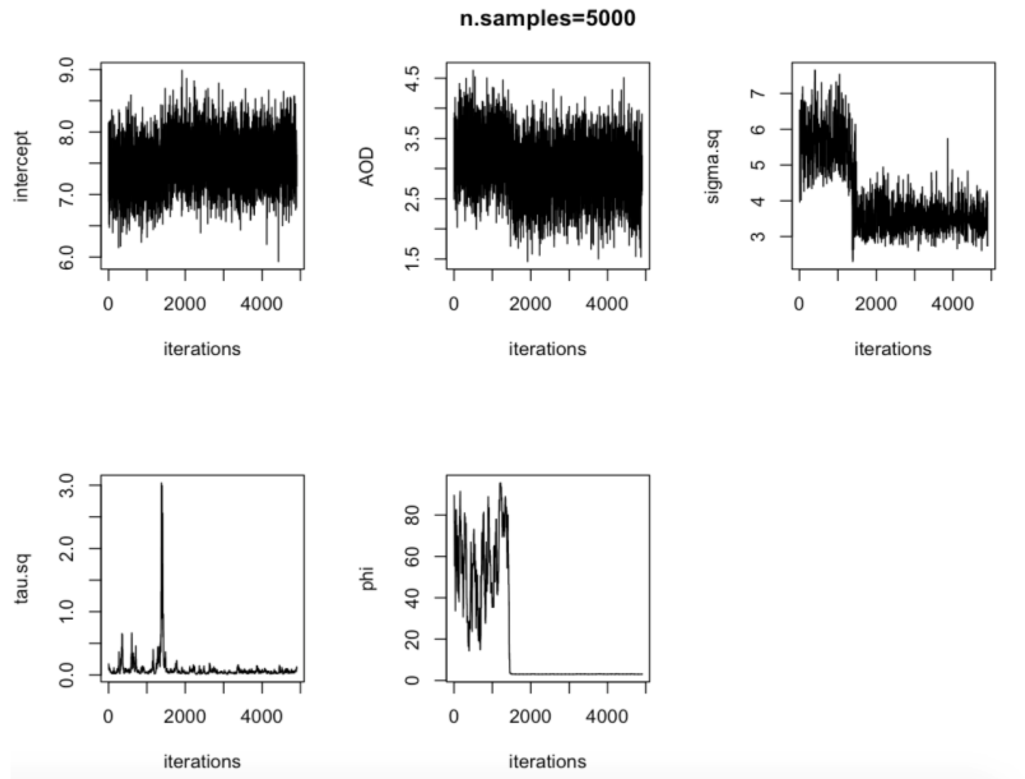

Figure S2. Trace plots of all the parameters after 5,000 iterations.

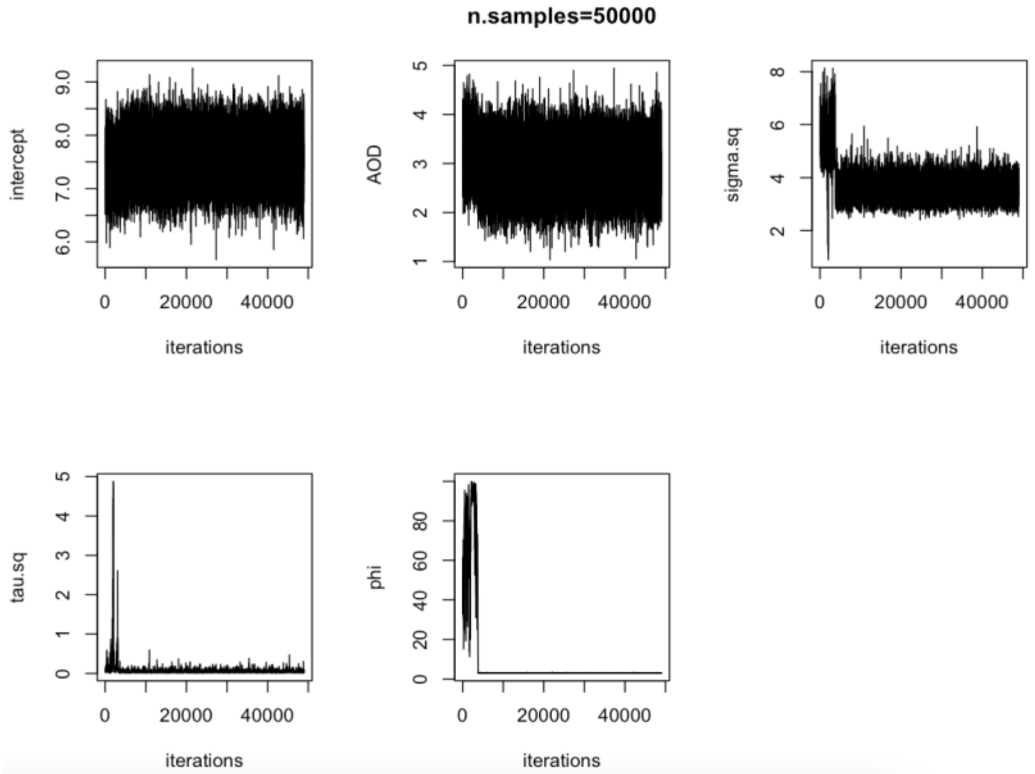

Figure S3. Trace plots of all the parameters after 50,000 iterations.

In addition, Heidelberger and Welch's test was used for all the daily models to determine whether these parameters had converged after 5,000 iterations. An annual convergence rate was calculated as the ratio of the number of days on which convergence was achieved to the total number of days (365; Table S4).

Table S4. Annual convergence rate for parameters

Parameter	$\boldsymbol{\beta_0}$	$\boldsymbol{\beta_1}$	$\boldsymbol{\sigma^2}$	$\boldsymbol{\tau^2}$	$\boldsymbol{\phi}$
Annual convergence rate (%)	97.5	94.8	90.1	0	80.8

We note that 5,000 iterations should sufficient to obtain converged estimates for most of the parameters (β_0, β_1, $\sigma^2$and ϕ). However, the value of τ^2 is very small, making it sensitive to changes; thus, it converges with difficulty. We thus selected a run length of 5,000 iterations and a burn-in time of 3,000 iterations.

Table S5. Model Fitting and cross-validation results of the Gaussian processes, GWR and LME models

	Model fitting		Cross-validation	
	R^2	RMSE (μg/m^3)	R^2	RMSE (μg/m^3)
Gaussian processes	1.00	0.01	0.81	21.87
GWR	0.86	20.05	0.74	25.71
LME	0.49	30.17	0.48	30.38
